# Comparison of the effects of direct and remote ischemic post and preconditioning in the kidney of young rats: histological and laboratory evaluation

**DOI:** 10.1590/acb410726

**Published:** 2026-02-06

**Authors:** Arthur Henrique de Almeida Oliveira, Daniel Meirelles de Paula, Suellen Serafini, Uenis Tannuri, Ana Cristina Aoun Tannuri

**Affiliations:** 1 Universidade de São Paulo – Faculdade de Medicina – Departamento de Pediatria – São Paulo (SP) – Brazil.; 2 Universidade de São Paulo – Faculdade de Medicina – Laboratório de Investigação Médica em Cirurgia Pediátrica – São Paulo (SP) – Brazil.

**Keywords:** Kidney, Reperfusion Injury, Ischemic Preconditioning, Ischemic Postconditioning, Rats

## Abstract

**Purpose:**

To evaluate the effects of direct and remote ischemic preconditioning and postconditioning on kidney ischemia–reperfusion injury in young rats.

**Methods:**

This study was divided into two experimental phases: preconditioning and postconditioning. In each phase, 78 rats were randomly assigned to four groups: control (CG), ischemia-reperfusion (IRG), direct ischemic pre/postconditioning (DIPG), and remote ischemic pre/postconditioning (RIPG). Animals were euthanized at 0- or 24-hours post-procedure, and samples were collected for histological and biochemical analyses.

**Results:**

The sum of the histological findings revealed no significant differences among the pre/postconditioning groups. However, serum calcium levels were significantly higher in preconditioning, DIPG compared to the control group (*p* = 0.002), and in postconditioning, DIPG and RIPG compared to the control group (*p* = 0.028 and *p* = 0.005, respectively). In postconditioning, the chlorine levels were higher in IRG compared to the DIPG group (*p* = 0.029).

**Conclusion:**

In our findings, ischemic preconditioning and ischemic postconditioning no provided significant protection of renal tissue.

## Introduction

Kidney transplantation is the recommended treatment for patients with end-stage renal disease (ESRD), whether they are undergoing dialysis or are in the pre-dialysis stage. It is also indicated in cases of acute kidney diseases such as glomerulonephritis, lupus nephropathy, vasculitis, trauma, or progressive glomerulosclerosis. In pediatric patients, impaired renal function may also result from congenital malformations of the urinary tract^1^.

In children, transplantation is the treatment of choice for end-stage chronic kidney disease. Despite the inherent risks associated with surgery and the postoperative period, kidney transplantation significantly improves both survival rates and quality of life in pediatric patients^1^^,^ 2.

Since the first successful kidney transplant in 1954, numerous studies have focused on optimizing surgical techniques and postoperative management. However, several biological challenges remain, from organ procurement to implantation. Among these, ischemia-reperfusion injury (IRI) is one of the most critical. IRI is a pathological process characterized by a temporary interruption of blood supply followed by subsequent reperfusion and reoxygenation, which can cause cellular damage^1^^–^ 3.

IRI can severely compromise graft function, increasing the risk of acute rejection and negatively affecting long-term transplant outcomes. To mitigate these effects, some studies have proposed ischemic conditioning techniques. These involve brief and alternating cycles of ischemia and reperfusion applied to tissues, aiming to reduce the severity of IRI.

Such models include ischemic preconditioning—applied prior to prolonged ischemia—, either directly to the target organ or remotely to a different tissue or organ. Ischemic postconditioning, on the other hand, is performed after the ischemic event, also using brief ischemia and reperfusion (IR) cycles to promote tissue protection^3^.

Between 2016 and 2020, 1,509 pediatric kidney transplants were performed in Brazil^4^. However, the literature lacks experimental models of ischemic preconditioning and postconditioning specifically designed for young rats, which are physiologically closer to pediatric patients than adult animal models. Therefore, this study was designed to establish and evaluate preconditioning and postconditioning protocols in young rats subjected to IRI, with the goal of translating these findings into improved outcomes in pediatric kidney transplantation.

## Methods

This research followed the protocols in the guidelines for the care and use of laboratory animals prepared by the National Academy of Sciences. The study protocol was reviewed and approved by the Animal Ethics Committee (CEUA) at our institution (Universidade de São Paulo, Medical School, São Paulo, SP, Brazil), CEUA number 1,410/2019.

A total of 78 newly weaned Wistar rats were used, between 20 and 30 days old, of both sexes, weighing 70–100 g and measuring 22–26 cm, equally distributed between groups (of six animals each) as demonstrated bellow:

- Control group (CG): six rats;
- Preconditioning groups 0 h:
  - Ischemia-reperfusion group (IRG): six rats;
  - Direct ischemic preconditioning (DIPG): six rats;
  - Remote ischemic preconditioning (RIPG): six rats.
- Preconditioning groups 24 h:
  - Ischemia-reperfusion group (IRG): six rats;
  - Direct ischemic preconditioning (DIPG): six rats;
  - Remote ischemic preconditioning (RIPG): six rats.
- Postconditioning groups 0 h:
  - Ischemia-reperfusion group (IRG): six rats;
  - Direct ischemic preconditioning (DIPG): six rats;
  - Remote ischemic preconditioning (RIPG): six rats.
- Postconditioning groups 24 h:
  - Ischemia-reperfusion group (IRG): six rats;
  - Direct ischemic preconditioning (DIPG): six rats;
  - Remote ischemic preconditioning (RIPG): six rats.

Before surgery, the animals were anesthetized with isoflurane (Isothane) by inhalation until sedation, followed by intramuscular doses of 1 mL/100 g of ketamine hydrochloride (Ketalar) and 10 mg/kg of Precedex (dexmedetomidine).

In the CG, six rats underwent left nephrectomy via midline laparotomy. In the IRG pre, 12 rats initially underwent left unilateral nephrectomy and then IRI (30 minutes of ischemia followed by 30 minutes of reperfusion) in the animal’s right kidney through clamping and subsequent declamping of the renal pedicle. For euthanasia and kidney removal, these rats were subdivided into two groups, with six of them having their kidneys removed immediately after the completion of the reperfusion period (group 0 h) and the six remaining were removed 24 hours after the IRI (group 24 h).

In the DIPG pre, 12 rats were initially submitted to left unilateral nephrectomy and then submitted to the DIPG protocol, which consists of ischemia of the right renal pedicle for 5 minutes followed by reperfusion for 5 minutes, using clamping and unclamping. This cycle was performed three times. After performing the three cycles of the DIPG preconditioning protocol, an IRI, as previously described, was performed in the animal’s right kidney by clamping and subsequently declamping the renal pedicle. For euthanasia and kidney removal, these rats were subdivided into two groups, 0 h and 24 h.

**Figure 1:**
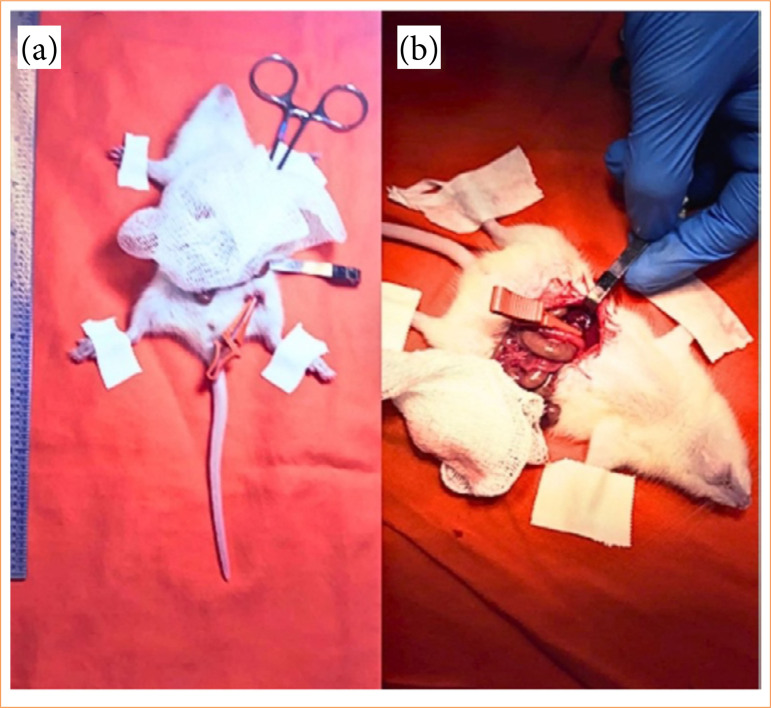
Animals submitted to ischemic conditioning: **(a)** remote ischemic preconditioning rat; **(b)** direct ischemic preconditioning rat.

Source: Elaborated by the authors.

In the RIPG pre, 12 rats were initially submitted to unilateral left nephrectomy and then submitted to the RIPG preconditioning protocol, which consists of ischemia of the left hind limb for 5 minutes followed by reperfusion for 5 minutes, through clamping and subsequent declamping of the femoral artery. This cycle was performed three times. After performing the three cycles of the RIPG preconditioning protocol, IRI was made in the right kidney by clamping and subsequently declamping the renal pedicle. For euthanasia and kidney removal, these rats were subdivided into two groups, 0 h and 24 h (Fig. 1).

For the postconditioning groups, we used the same methodology from the preconditioning groups. The difference between them was the moment at which the IRI conditioning cycles occurred; in the preconditioning groups, the cycles occurred before ischemic injury, whereas in the postconditioning groups they occurred immediately after 30 minutes of the reperfusion.

### Biochemical parameters

In all groups, blood was collected during euthanasia, prior to cardiac arrest, via aortic puncture. Blood samples were drawn, and the serum was separated by centrifugation at 4,000 rpm for 10 min at 4°C, to determine urea, creatinine, sodium, calcium and chlorine levels.

### Histological analysis

The kidneys were removed during euthanasia and performed by paraffine. Using the cuts from the paraffined material, histological analyses were performed. Five-µm sections were stained with hematoxylin and eosin. After proper staining, the slides were photographed. The assessment of the degree of tubulointerstitial lesion was performed blindly and semiquantitatively via scores from 0–4:

- 0: absence of alteration;
- 1: less than 5% lesion;
- 2: 5–25% lesion;
- 3: 25–75%;
- 4: above 75% in each field.

For each slide, five fields were analyzed; one field is equivalent to the area covered by th e 40X zoom.

A total of five histological findings were analyzed:

- Tubular epithelial lesion;
- Brush border loss;
- Cylinders and cell debris;
- Focal tubular necrosis;
- Edematous degeneration^5-7^.

A score was used to condense all the parameters, and all the findings had the same weight. To calculate the sum of histological changes, we added all the values attributed and their corresponding variable, obtaining a maximum theoretical score of 20.

### Statistical analysis

Statistical analysis was carried out using Statistical Package for the Social Sciences version 18.0 for Windows (Chicago, United States of America). The Shapiro-Wilk’s test was used to determine whether groups of data had a Gaussian distribution. The significance of differences among groups was examined using analysis of variance (ANOVA) and Tukey’s post hoc test for parametric data or Kruskal-Wallis’ test and Dunn’s post hoc test with Benjamini-Hochberg *p*-value correction method for non-parametric data. *p* < 0.05 (two-tailed) were considered significant.

## Results

### Histological analysis

#### Preconditioning

**Figure 2:**
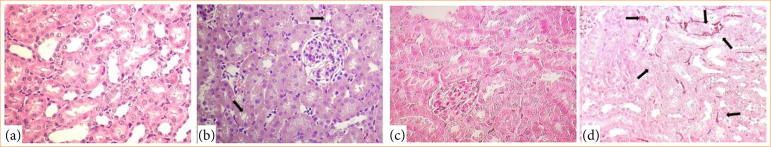
Hematoxylin and eosin visualization of the renal parenchyma. **(a)** Renal tubules without alteration. **(b)** Hydropic degeneration of some cells of the proximal tubules, arrows point to loss of brush border. **(c)** Intense hydropic degeneration and tubular necrosis. **(d)** Arrows point to presence of cylinders and cell debris. Scale: 400x.

Source: Elaborated by the authors.

Histological analysis revealed significant differences between groups in two out of the five evaluated parameters: epithelial lesion, and tubular necrosis (Fig. 2).

For the epithelial lesion, a significant reduction was observed in the RIPG 0 h compared to the IR and DIPG groups (*p* = 0.042 and 0.031, respectively).

At 0 h, hydropic degeneration score was higher in both IR and DIPG than CG (*p* = 0.004 and 0.031, respectively). At 24 h, hydropic degeneration was more pronounced in IRG and RIPG groups than in CG (*p* = 0.007 and 0.013).

**Figure 3:**
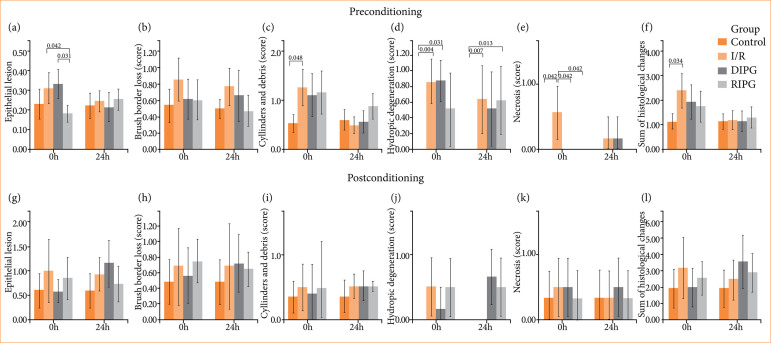
Graphs showing the values obtained with the histological analysis.

Source: Elaborated by the authors.

Tubular necrosis was significantly more pronounced in IRG compared to CG 0 h (*p* = 0.042). Both DIPG 0 h and RIPG 0 h showed significantly lower scores than IRG 0 h (*p* = 0.042 for both) (Fig. 3).

#### Postconditioning

The histological analysis showed no significant differences between the IRG and the groups subjected to the conditioning technique, regardless of the reperfusion time (0 or 24 hours) (Fig. 3).

### Biochemical analysis

In the serum analysis, calcium levels were significantly higher in the DIPG 0h compared to the CG (*p* = 0.002). Similarly, in the postconditioning phase, both the DIPG 0 h and RIPG 0 h groups showed elevated serum calcium levels compared to the CG (*p* = 0.009 and *p* = 0.034, respectively)

**Figure 4:**
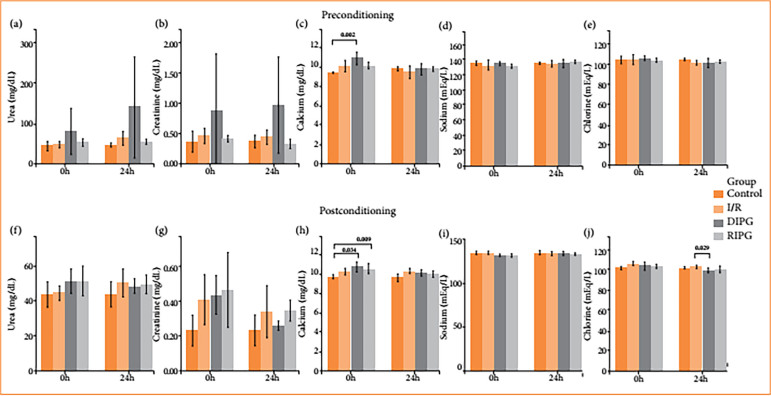
Graphs showing the values obtained with the histological analysis.

Source: Elaborated by the authors.

The chlorine level in DIPG 24 h was lower when compared with IRG 24 h (Fig. 4).

## Discussion

Due to the limited availability of data on ischemic preconditioning and postconditioning techniques in the current medical literature, this study was conducted with the aims of contributing foundational knowledge and guiding future research on the application of these techniques in pediatric populations, using a young animal model.

Given that transplantation is considered the most effective therapy for children with ESRD, the development of experimental models that more closely resemble pediatric patients is of great value for advancing strategies to mitigate IRI^4^^,^ 8.

Although several studies in adult rats have demonstrated significant reductions in IRI through conditioning techniques, very few have employed young rats as experimental models. The use of juvenile animals presents specific challenges, including the small size of organs and blood vessels, which complicates surgical procedures, and the difficulty in accurately titrating anesthetic doses in younger animals^9^^,^ 10.

In our model, we performed a contralateral nephrectomy prior to initiating the IRI cycles to enhance the sensitivity and impact of the intervention^11^.

The histopathological scoring system used in this study was adapted from previous literature, as no standardized or validated scoring system for renal IRI currently exists^3^^,^ 11.

Previous studies investigating ischemic conditioning techniques—both pre- and postconditioning, and in both direct and remote modalities—have reported improved outcomes in tissues subjected to IRI^12^^–^ 14. These findings support the potential clinical relevance of such strategies, particularly in vulnerable populations such as pediatric transplant recipients.

### Preconditioning

Our histological findings indicated that, soon after IRI, remote ischemic preconditioning led to a reduction in the epithelial lesion and hydropic degeneration. Besides, in relation to tubular necrosis, both direct and remote preconditioning groups showed better results compared to IRI group.

These results suggested that, even in young organisms, there is a protective effect when preconditioning is performed, and they align with previous studies reporting that ischemic preconditioning can mitigate histological features such as hydropic degeneration and necrosis^3^.

Overall, both remote and direct ischemic preconditioning techniques tended to show beneficial in reducing total histological damage, even though this reduction did not present a statistically significant difference.

Our findings are consistent with other published studies in the field^3^^,^ 9 ^,^ 12 ^,^ 15 ^,^ 16.

In contrast, the serum markers typically used to assess acute kidney injury—urea and creatinine—did not correlate with the histological findings. These markers failed to reflect the degree of tissue damage observed microscopically, highlighting a limitation in their sensitivity during the early stages of injury.

However, serum calcium levels showed a statistically significant difference between the DIPG 0 h and the CG, suggesting that calcium may serve as an early biomarker of renal dysfunction. As illustrated in Fig. 4, ischemic injury appears to impair the kidney’s ability to regulate calcium homeostasis immediately after reperfusion. In contrast, calcium levels in the 24-h groups returned to values comparable to those of the CG, indicating that renal function can recover substantially within this timeframe, even in the context of considerable ischemic insult. Similar patterns of transient hypercalcemia following ischemic preconditioning have been reported in previous studies.

As for sodium and chloride levels, no significant differences were observed among the groups. This indicates that sodium balance is maintained despite ischemic injury, and given the close physiological coupling between sodium and chloride transport, chloride levels also remained within normal ranges.

### Postconditioning

Histological analysis yielded results that did not support the efficacy of the postconditioning technique, showing no significant reduction in tissue injury. There were no statistically significant differences between the IRI group and the postconditioning groups, regardless of the reperfusion time applied (30 minutes or 24 hours).

Similarly, the biochemical analyses did not support a protective effect of renal postconditioning. Serum calcium levels were significantly elevated in both the direct (DIPG 0 h) and remote (RIPG 0 h) postconditioning groups compared to the CG, likely reflecting increased cellular injury in animals subjected to the technique. These findings further contradict the anticipated benefits of postconditioning. Among the biochemical parameters, chloride was the only one to show a significant reduction, observed in the comparison between the IRG 24 h and DIPG 24 h.

Overall, postconditioning failed to demonstrate protective effects when compared to the CG, in contrast to several published studies. For example, Yamaki et al.^3^ and van den Akker et al.^13^ reported beneficial outcomes associated with the use of ischemic postconditioning. Several factors may account for this discrepancy, including variations in ischemia duration, blood sampling techniques, or experimental protocols.

One notable methodological difference in our study was the performance of nephrectomy prior to the induction of ischemia, a step not commonly included in other models. Additionally, in remote postconditioning groups, manipulation of the femoral artery may have caused muscle injury, potentially influencing serum creatinine and urea levels and confounding the results.

It is also important to consider that our model employed young rats, whereas most previous studies were conducted on adult animals. Age-related differences in physiological responses to ischemic conditioning may have contributed to the divergent outcomes observed.

A potential limitation of our study is the timing of sample collection and analysis. We chose a 24-hour post-reperfusion time point, which may have been insufficient to observe the full protective effects of postconditioning. Some studies have reported more favorable outcomes at 48 hours^12^^,^ 17 ^,^ 18.

Although existing literature generally supports the efficacy of ischemic preconditioning and postconditioning, a lack of standardization regarding critical aspects such as the type and duration of conditioning, histopathological evaluation criteria, and animal models used remains. These inconsistencies highlight the need for further studies to establish uniform protocols and improve comparability across research in this field^10^^,^ 13 ^,^ 14.

## Conclusion

The preconditioning technique exhibited a degree of renal protection at the histological level when compared to postconditioning. While most laboratory parameters did not show statistically significant differences among the groups, serum analyses revealed higher markers of acute kidney injury in the postconditioning groups relative to both the preconditioning and CG.

## Data Availability

All data sets were generated or analyzed in the current study.

## References

[1] Bonthuis M, Harambat J, Jager KJ, Vidal E (2021). Growth in children on kidney replacement therapy: a review of data from patient registries. Pediatr Nephrol.

[2] Hebert SA, Swinford RD, Hall DR, Au JK, Bynon JS (2017). Special considerations in pediatric kidney transplantation. Adv Chronic Kidney Dis.

[3] Yamaki VN, Gonçalves TB, Coelho JV, Pontes RV, Costa FL, Brito MV (2012). Protective effect of remote ischemic per-conditioning in the ischemia and reperfusion-induced renal injury in rats. Rev Col Bras Cir.

[4] Faro BL, Lima BM, Nogueira HG, Barreto ICR, Santos RGA, Mendonça VPV, Bispo AJB, Santos AS (2022). Kidney transplantation and its relation with growth in the pediatric age group: a bibliographic review. Braz J Health Rev.

[5] Shanley PF, Rosen MD, Brezis M, Silva P, Epstein FH, Rosen S (1986). Topography of focal proximal tubular necrosis after ischemia with reflow in the rat kidney. Am J Pathol.

[6] Xu L, Sharkey D, Cantley LG (2019). Tubular GM-CSF promotes late MCP-1/CCR2-mediated fibrosis and inflammation after ischemia/reperfusion injury. J Am Soc Nephrol.

[7] Castro AF, Castro LPF, Leite VHR, Paulino E, Lima AS, Gazzola L, Toppa NH (2002). Achados histológicos em 48 pacientes transplantados do fígado: biópsias do enxerto pós-reperfusão (tempo zero) e de três a 15 dias pós-transplante. J Bras Patol Med Lab.

[8] Sözen H, Dalgic A, Karakayali H, Baskin E, Saatci U, Arslan G, Haberal M (2006). Renal transplantation in children. Transplant Proc.

[9] Ortega-Trejo JA, Bobadilla NA (2023). Is renal ischemic preconditioning an alternative to ameliorate the short- and long-term consequences of acute kidney injury?. Int J Mol Sci.

[10] Jonker SJ, Menting TP, Warlé MC, Ritskes-Hoitinga M, Wever KE (2016). Preclinical evidence for the efficacy of ischemic postconditioning against renal ischemia-reperfusion injury, a systematic review and meta-analysis. PLoS One.

[11] Zhang WX, Yin W, Zhang L, Wang LH, Bao L, Tuo HF, Zhou LF, Wang CC (2009). Preconditioning and postconditioning reduce hepatic ischemia-reperfusion injury in rats. Hepatobiliary Pancreat Dis Int.

[12] Wever KE, Menting TP, Rovers M, van der Vliet JA, Rongen GA, Masereeuw R, Ritskes-Hoitinga M, Hooijmans CR, Warlé M (2012). Ischemic preconditioning in the animal kidney, a systematic review and meta-analysis. PLoS One.

[13] van den Akker EK, Manintveld OC, Hesselink DA, de Bruin RW, Ijzermans JN, Dor FJ (2013). Protection against renal ischemia-reperfusion injury by ischemic postconditioning. Transplantation.

[14] Giannopoulos G, Vrachatis DA, Panagopoulou V, Vavuranakis M, Cleman MW, Deftereos S (2017). Remote ischemic conditioning and renal protection. J Cardiovasc Pharmacol Ther.

[15] Kalogeris T, Baines CP, Krenz M, Korthuis RJ (2012). Cell biology of ischemia/reperfusion injury. Int Rev Cell Mol Biol.

[16] Cochrane J, Williams BT, Banerjee A, Harken AH, Burke TJ, Cairns CB, Shapiro JI (1999). Ischemic preconditioning attenuates functional, metabolic, and morphologic injury from ischemic acute renal failure in the rat. Ren Fail.

[17] Jankauskas SS, Pevzner IB, Andrianova NV, Zorova LD, Popkov VA, Silachev DN, Kolosova NG, Plotnikov EY, Zorov DB (2017). The age-associated loss of ischemic preconditioning in the kidney is accompanied by mitochondrial dysfunction, increased protein acetylation and decreased autophagy. Sci Rep.

[18] Zhuang S, Lu B, Pang M (2009). Postconditioning does not improve renal function or attenuate tubular damage in ischemia/reperfusion-induced acute kidney injury in mice. Open Pathol J.

